# tRNA-derived small RNAs: novel regulators of cancer hallmarks and targets of clinical application

**DOI:** 10.1038/s41420-021-00647-1

**Published:** 2021-09-18

**Authors:** Xizhe Li, Xianyu Liu, Deze Zhao, Weifang Cui, Yingfang Wu, Chunfang Zhang, Chaojun Duan

**Affiliations:** 1 Department of Thoracic Surgery, Xiangya Hospital, Central South University, Xiangya Road 87th, Changsha, 410008 Hunan P. R. China; 2 Hunan Engineering Research Center for Pulmonary Nodules Precise Diagnosis & Treatment, Changsha, 410008 Hunan P. R. China; 3 Centre of Stomatology, Xiangya Hospital, Central South University, Xiangya Road 87th, Changsha, 410008 Hunan P. R. China; 4 National Clinical Research Center for Geriatric Disorders, Changsha, 410008 Hunan P. R. China; 5 Institute of Medical Sciences, Xiangya Lung Cancer Center, Xiangya Hospital, Central South University, Changsha, 410008 Hunan P. R. China

**Keywords:** Cancer epigenetics, Tumour biomarkers

## Abstract

tRNAs are a group of conventional noncoding RNAs (ncRNAs) with critical roles in the biological synthesis of proteins. Recently, tRNA-derived small RNAs (tsRNAs) were found to have important biological functions in the development of human diseases including carcinomas, rather than just being considered pure degradation material. tsRNAs not only are abnormally expressed in the cancer tissues and serum of cancer patients, but also have been suggested to regulate various vital cancer hallmarks. On the other hand, the application of tsRNAs as biomarkers and therapeutic targets is promising. In this review, we focused on the basic characteristics of tsRNAs, and their biological functions known thus far, and explored the regulatory roles of tsRNAs in cancer hallmarks including proliferation, apoptosis, metastasis, tumor microenvironment, drug resistance, cancer stem cell phenotype, and cancer cell metabolism. In addition, we also discussed the research progress on the application of tsRNAs as tumor biomarkers and therapeutic targets.

## Facts

- tsRNAs are a novel group of ncRNAs that have critical biological functions through translation modification, miRNA-like functions, and their interaction with PIWI proteins.
- tsRNAs are widely and abnormally expressed in tumor tissues and can regulate the biological hallmarks of cancer cells.
- The application of tsRNAs as biomarkers and therapeutic targets of cancer is an emerging topic in cancer research.

## Questions

- How are all these types of tsRNAs cleaved by ribonucleases?
- What is the general pattern of biological functions of tsRNAs that are involved with proteins other than AGOs?
- What are the exact functions of tsRNAs in the tumor microenvironment?

## Introduction

Noncoding RNAs (ncRNAs) that are not translated exist widely in mammalian cells. Small ncRNAs (sncRNAs) are a group of ncRNAs up to 200 nucleotides (nt) in length that include microRNAs (miRNAs), small interfering RNAs (siRNAs), and small nucleolar RNAs (snoRNAs), and have various biological functions in multiple cellular processes. These sncRNAs have been shown to mediate multiple biological functions by RNA silencing, translation regulation, and many other molecular mechanisms.

Transfer RNAs (tRNAs) are a group of classic ncRNAs and are well known for the translation of amino acids to messenger RNAs (mRNAs) [1]. In addition to protein synthesis, tRNAs are also involved in cell proliferation and tumorigenesis [2]. Mature tRNAs are ~70–90 nt with a D-loop, a TψC loop, an anti-codon loop, a variable loop, and an acceptor arm. There are many lines of evidence demonstrating that the dysregulation of genes is related to the posttranscriptional modification of tRNA, and cytoplasmic tRNA-related proteins are associated with human diseases [3].

With the breakthrough of high-throughput sequencing and microarray technologies, abundant novel small ncRNAs types were found. In 1979, tRNA-derived small RNAs (tsRNAs) were first found in cancer patients [4]. tsRNAs were initially considered as random degradation products during tRNA biogenesis and degradation. However, gradually accumulating evidence has shown that tsRNAs have few relationships with parental tRNA abundance. They are produced by specific nucleases at the specific site of mature or pre-tRNAs and are closely related to metabolism, viral infection, neurodegeneration, and tumorigenesis [5–11]. Like miRNAs, tsRNAs were widely detected in cells from most types of cancer tissues. Recent studies showed that tsRNAs play crucial roles in cell proliferation, apoptosis, metabolism, metastasis, and other characteristics of most cancer types by participating in various molecular processes such as protein synthesis, gene silencing, and RNA processing. Meanwhile, tsRNAs have attracted much attention because of their potential application as biomarkers and therapeutic targets of tumor diseases. Therefore, these findings demonstrate that tsRNAs can be new regulatory factors for the hallmarks of cancer.

In this review, we will discuss the characteristics of tsRNAs, dysregulation of tsRNAs in tumor cells, and explore the regulatory roles of tsRNAs in cancer biological functions. In addition, we will also discuss the application opportunity of tsRNAs as tumor biomarkers and therapeutic targets.

## Classification and biogenesis of tsRNAs

**Fig. 1:**
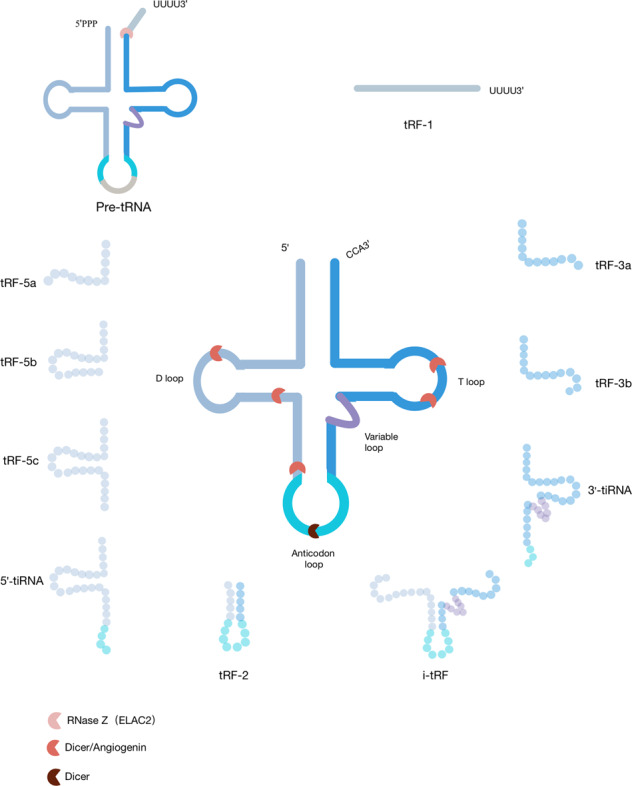
Classification of tsRNAs. tRF-1s are derived from the 3′-end of the precursor tRNA. tRF-5s are derived from the 5′-end of mature tRNAs and end at a D-loop or at a stem region between the D-loop and the anti-codon loop. tRF-3s are generated from the TψC loop with a “CCA” tail. tRF-2s are produced by decomposition of the anticodon loop of tRNA and do not include the typical 5′-end and 3′-end groups. i-tRFs are generated from the internal region of mature tRNAs straddling the anti-codon region with variable length. tiRNAs are almost half the length of mature tRNAs.

In recent years, many researchers focused on tsRNA-related studies. However, these researchers promoted their own nomenclature for tsRNAs without a unified principle. Therefore, the nomenclature of tsRNAs is confusing and redundant. Herein, we will discuss the main classifications of tsRNAs, and elucidate the relationship between these subtypes. tsRNAs are cleaved from precursor or mature tRNAs with a length of 18–40 nt and include two major components: tRNA halves and tRNA-related small RNA fragments (tRFs). tRNA halves are longer at 30–40 nt. tRFs are shorter and have a length of 18–30 nt (Fig. 1). Meanwhile, researchers suggest that all these types of tsRNAs are closely intertwined with mRNAs in cancer cells [12].

tRNA halves were first described in 1969, and named because their structure is almost half that of mature tRNAs [13]. They are more commonly called tRNA-derived stress-induced RNAs (tiRNAs) because their biogenesis largely occurs under conditions of stress such as hypoxia, oxidative stress, heat shock and nutritional deficiency [14, 15]. Mature tRNAs are cleaved into 5′-tRNA halves (5′-tiRNAs) and 3′-tRNA halves (3′-tiRNAs) at the anticodon loops by angiogenin (ANG). 5′-tiRNAs are cleaved from the 5′-end of mature tRNAs to the end of the anticodon loop, and 3′-tiRNA starts at the nucleotide in the anti-codon loop and proceeds to the 3′-ends of mature tRNAs [16].

tRFs consist of tRF-5s, tRF-3s, tRF-1s (3′U-tRFs), and tRF-2s (i-tRFs). tRF-5s, tRF-3s, and tRF-2s are the products of mature tRNA cleavages, and tRF-1s are cut from pre-tRNAs. A tRF-5s (14–30 nt) is derived from the 5′-end of a mature tRNA and ends in the D-loop (tRF-5a) or at a stem region between the D-loop and the anti-codon loop (tRF-5b and tRF-5c) of mature tRNAs. They can be classified into three specific lengths, tRF-5a (14–16 nt), tRF-5b (22–24 nt), and tRF-5c (28–30 nt) [17]. A tRF-3s is generated from the TψC loop to the “CCA” tail and mainly contains two subtypes: tRF-3a and tRF-3b. The difference between these two subtypes is the sequence length. Members of the tRF-3b family are ~4 nt longer than members of the tRF-3a family and can be 22-nt long [18]. Previously, tRF-5s and tRF-3s were identified as Dicer-dependent products, whereas further studies found that ANG and other members of the ribonuclease A superfamily also participate in the cleavage of these tRFs [17, 19].

tRF-1s (16–48 nt) are derived from the 3′-end of the precursor tRNA in the nucleus by the ribonuclease Z (RNaseZ) or in the cytoplasm by the homologous ribonuclease Z2 (ELAC2) [20, 21]. Both tRF-1s and tRF-3s have the 3′-end of tRNAs, but have diverse biological functions because of the difference in 5′-ends [22]. The length of tRF-1s is more diverse than that of other tRFs because of the various positions that endonucleases recognize. Unlike the tRFs described above, tRF-2s (i-tRFs) are a newly discovered type of tRF that are generated from the internal region of mature tRNAs straddling the anti-codon region with variable length [23]. Thus far, the synthesis of tRF-2s and the ribonucleases that participate in this process are still unclear.

Researchers also name the tsRNAs in studies according to their functional mechanisms. For example, in Honda’s study, the authors defined the abundantly expressed tRNA halves in estrogen receptor (ER)-positive breast cancer and androgen receptor (AR)-positive prostate cancer cell lines as Sex Hormone-dependent tRNA-derived RNAs (SHOT-RNAs) [24]. Meanwhile, another study identified PIWI-interacting tsRNAs as td-piRs [25]. Although the nomenclature of tsRNAs is confusing, there are emerging online databases that summarize and name the validated tsRNAs, such as tRFdb (http://genome.bioch.virginia.edu/trfdb/) and OncotRF (http://bioinformatics.zju.edu.cn/OncotRF) [6, 26].

## Biological functions of tsRNAs

**Fig. 2:**
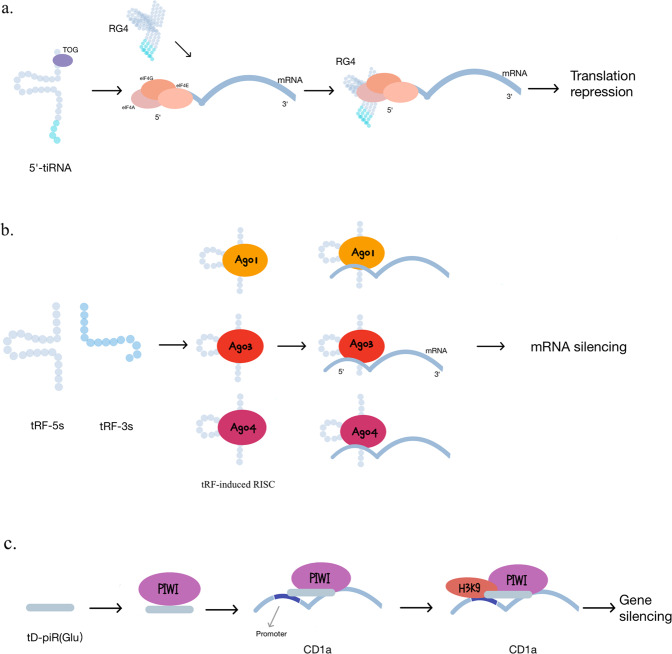
Biological functions of tsRNAs. **a** The oligoguanine sequence at the 5′ terminus (TOG) of the 5′-tiRNA can be assembled into an RNA G-quadruplex (RG4) structure and then leading to translation repression by replacing eIF4F complex with mRNA. **b** tRF-3s and tRF-5s tend to associate with Ago 1, 3, and 4 and form tRF-induced RISCs. They target the coding region, 3′ UTRs and 5′ UTRs of mRNA. **c** tD-piR (Glu) can bind the PIWIL4 protein to form a complex and then recruit H3K9 methyltransferases to the CD1a (a specific and functional marker of DCs) promoter region. Highly expressed td-piR(Glu) inhibits CD1a transcription in monocytes.

The first identified biological function of tsRNAs was gene expression inhibition through miRNA-like silencing. However, recent studies have shown that tsRNAs can bind proteins other than AGOs and exhibit more complicated molecular functions. Herein, we will introduce the three major tsRNA-induced molecular mechanisms (Fig. 2).

### Translation modulation

tsRNAs are thought to repress mRNA translation in *Haloferax volcanii*, in which a subset of 26 nt tRF-5s play significant roles [27]. A study showed that the conserved residues in tRNAs were critical components for the appropriate 3-dimensional folding and recognition by the translation machinery [22]. In a subsequent study, Sobala et al. found that tRF-5s contained a conserved GG dinucleotide sequence necessary for translation inhibition [28].

Base modification is one of the vital determinants of the biogenesis and function of tsRNAs. The cytosine-5 RNA methyltransferase NSUN2 is highly expressed in epithelial tumors. The loss of cytosine-5 RNA methylation (m5c) due to NSUN2 deficiency promotes the ANG-induced cleavage of tRNA and leads to an accumulation of 5′-tRNA halves. Meanwhile, the accumulated 5′-tRNA halves in Nsun2−/− cells inhibit mRNA translation rates, promote the activation of stress pathways, decrease cell size and increase cell death [29]. Similarly, Ivanov et al. revealed that 5′-tiRNAs impede protein translation in osteosarcoma cells by displacing the initiation factors eIF4G/eIF4A and eIF4F from mRNA [30]. Another study by Emara et al. revealed that 5′-tiRNAs induced by ANG repressed translation initiation and promoted the phospho-eIF2α-independent assembly of stress granules (SGs) [31]. In addition, Guzzi et al. also found that tRF-5s with TOG, such as tRNA-Ala, tRNA-Cys, and tRNA-Val were significantly absent in PUS7-KO cells, and these tRFs mediated protein biosynthesis and defective germ layer specification in embryonic stem cells. Moreover, the pseudouridine writer PUS7 activated this tRF-mediated translation regulation [32]. In addition, Lyons and his colleagues also demonstrated that RNA G-quadruplex (RG4) structures are required for the translation regulatory functions of tsRNAs [33].

### miRNA-like functions

It has been suggested that some tRFs have a similar 5′ phosphate and a 3′ hydroxyl group as microRNAs (miRNAs) and perform analogous effects [34]. Previous studies suggested that there is cross-mapping between miRNA precursors and tRFs, and conventionally defined mature miRNAs also overlap with tRFs. tRFs associate with Ago by cross-linking the seven base seed sequence at the 5′ end of tRFs and then form a complex with Ago in a tRF-target-Ago manner, which is similar to the behavior of the miRNA-derived RNA-induced silencing complex (RISC) [35]. After mutation of the complementary base pairing region of the target, the original effect of tRF-induced luciferase reporter repression was weakened [36]. The length of the tRF-induced RISC is approximate to the processed miRNA sequence because of the distinct three-dimensional structural performance of tRNAs [37]. These lines of evidence suggest that tRFs may be involved in gene-silencing through a miRNA-like mechanism. However, tRF-3s and tRF-5s tend to associate with Ago 1, 3, and 4, but not Ago 2, which is the main effector protein of miRNA-induced RISC [35]. Guan et al. found that tRFs break the conventional 3′ untranslated region (UTR) targeting pattern of miRNAs and target the coding region, 3′UTRs and 5′UTRs of mRNAs [38]. Furthermore, tRFs probably lead Ago to associate with the 5′ ends of agotrons (introns of mRNA) [38].

DICER is a crucial enzyme in miRNA biogenesis. It cleaves the loop in the stem-loop hairpin structure of the pre-miRNA to produce mature miRNAs [39]. After knockdown of DICER1, a tRF cloned from human mature B cells named CU1276 was reduced. This suggested that the Dicer-dependent cleavage step is a typical mechanism for tRF biogenesis [40]. However, in Dicer knockout cells, luciferase repression by tRF-3 remained [36]. This result suggested that Dicer cleavage may not be the only mechanism of tRF biogenesis.

### piRNA-like functions

tsRNAs can also participate in gene silencing by associating with the PIWI protein (PIWI) and act as piRNA (P-element-induced wimpy testis (PIWI)-interacting small RNAs) [41]. A key function of piRNAs is silencing transposon elements [42]. Ts-101 and ts-53 were reported to correlate with PIWI [5]. Compared with those in wild type controls, all members of the H3A1 histone subfamily were downregulated in HEK293-ts101-KO cells. Some tsRNAs from tRNA-Glu, tRNA-Gly, and tRNA-Pro have a similar length to classical piRNAs, so-called tD-piR. TD-piR (Glu) can bind the PIWIL4 protein to form a complex and then recruit H3K9 methyltransferases to the CD1a (a specific and functional marker of DCs) promoter region. In this way, highly expressed td-piR (Glu) enhances CD1a transcription in monocytes [25]. Moreover, a recent study revealed that tsRNAs could be loaded onto Ago proteins and play a role as miRNAs when one of the RBPs, lupus autoantigen (La), was absent [43].

## tsRNAs and cancer hallmarks

With a deeper understanding of tsRNA biological functions, the critical roles of tsRNAs in human diseases have attracted increasing attention. Recent studies have shown that tsRNAs participate in the development of metabolic and neurological diseases and have pivotal functions in vital infections and stress-induced cell damage. Not surprisingly, numerous studies have also revealed that tsRNAs can stimulate malignant progression and regulate tumor cell hallmarks. Herein, we summarized the biological cancer hallmarks that are certainly or potentially regulated by tsRNAs (Table 1).

**Table 1:** Roles of tsRNAs in cancer hallmarks.

tRF	Type	Hallmark	Cancer type	Regulation mechanism	Reference
tRF-Leu-CAG	tiRNA	Promote cell proliferation and cell cycle	NSCLC	Regulating AURKA	[45]
5′-tiRNAVal	tiRNA	Promote Progression and lymph node metastasis	Breast cancer	Suppresses the Wnt/β-catenin signaling pathway by targeting FZD3	[47]
ts-46 ts-47	tRF-1s	Inhibit cell proliferation	NSCLC	\	[5]
tRNA-Glu tRNA-Asp tRNA-Gly tRNA-Tyr	i-tRF	Inhibit cell proliferation and cancer metastasis	Breast cancer	YBX1 displacement	[57]
SHOT-RNAAsp-GUC SHOT-RNAHis-GUG SHOT-RNALys-CUU	tiRNA	Promote cell proliferation	Breast cancer Prostate cancer	\	[24]
tRF/miR-1280	/	Inhibit cell proliferation, migration, and cancer stem cells phenotype	Colorectal Cancer	Repressing Notch signaling pathways	[64]
LeuCAG3′tsRNA	tRF-3s	Promote cell apoptosis	Hepatocellular carcinoma	Binds two ribosomal protein mRNAs (RPS28 and RPS15) to enhance their translation	[61]
tRF-1001	tRF-1s	Promote cell proliferation	Prostate cancer	Accumulation of cells in the G2 phase of the cell cycle	[21]
CU1276	tRF-3s	Inhibit cell apoptosis	B cell lymphoma	Association with Argonaute protein and represses endogenous RPA1	[40]
ts-3676 ts-4521	tRF-3s	Tumor suppressor	Lung cancer CLL	Interacting with Piwi Proteins	[41]
ts-43 ts-44	tRF-5s	Tumor suppressor	CLL	\	[44]
tRF-03357	\	Promotes cell proliferation, migration, and invasion	High-grade serous ovarian cancer(HGSOC)	Modulating HMBOX1 in HGSOC	[52]
tRF5-Glu	tRF-5s	Inhibit cell proliferation	Ovarian cancer	Regulates BCAR3 expression	[53]
tRF-20-M0NK5Y93	tiRNA	Inhibit cell migration and invasion	Colorectal cancer	Targeting Claudin-1	[67]
tRF-315	tiRNA	Promote cisplatin resistance	Prostate cancer	Targeted the tumor suppressor gene GADD45A	[79]
tRF-3017A	tRF-3s	Promote the invasion and migration	Gastric cancer	Silencing Tumor suppressor NELL2	[69]

### Dysregulation of tsRNAs

tRF-1001 was the first tsRNA found to be abnormally expressed in a range of cancer cells [21]. After that, many dysregulated tsRNAs in tumor cells were also discovered. tRFs such as ts-3676 and ts-4521 are significantly downregulated and mutated in chronic lymphocytic leukemia (CLL) compared to normal controls [44]. CU1276, one of the tRF-3s, is highly expressed in mature B cells but absent in germinal center-derived lymphomas [40]. Shao et al. found that the expression of tRF-Leu-CAG was significantly upregulated in non-small cell lung cancer (NSCLC) tissues, cell lines and patient serum and positively correlated with tumor stage [45]. tDR-7816, a subtype of i-tRF, and 5′-tiRNA-Val were decreased in breast cancer cells [46, 47]. Intriguingly, 30–35 nt long 5′-tRNA halves were increased in chronic viral hepatitis but reduced in liver cancer tissues [48]. In silico analysis of the TCGA-BLCA project identified 5′-tRF-LysCTT as dysregulated in bladder tumors (BICa) [49]. It was significantly elevated in BICa and associated with an aggressive tumor phenotype, early disease progression, and a poor treatment outcome in 230 BICa patients. In addition, three 5′ tRFs were diversely expressed in testicular germ cell tumors compared with normal samples [50].

### Cell proliferation

As a well-known core hallmark, the proliferation capacity of cancer cells determines the progression of tumors and the prognosis of patients in a crucial way. In addition, this characteristic is the first cancer hallmark that was found to be associated with tsRNAs. tRF-1001 was the first tsRNA found to be associated with tumor proliferation. tRF-1001 is a prostate cancer susceptibility gene derived from pre-tRNA-Ser that is highly expressed in cancer cells and promotes cell proliferation by regulating the cell cycle [21].

Sex hormones play essential roles in the biogenesis of cancers, especially breast and prostate cancer. One newly identified type of tRF named SHOT-RNA produced by ANG is significantly elevated in estrogen receptor-positive breast cancer and androgen receptor-positive prostate cancer (PCa) cell lines. The cell growth rate was decreased when an siRNA targeting 5′SHOT-RNA was transfected with, and the levels of mature tRNAs were not changed in siRNA transfected cells [24]. These results suggested that SHOT-RNAs have an independent stimulating effect on cancer cell proliferation. Before this, Martens-Uzunova et al. found that 18-nt-long tRFs were significantly increased in PCa tissues by gene sequencing, whereas 27-nt-long tRFs were the most elevated tRFs [51].

tsRNAs also play a vital role in the proliferation of ovarian cancer cells. Small RNA sequencing and PCR results revealed that 27 tRFs were differentially expressed between high-grade serous ovarian cancer (HGSOC) patients and healthy control patients [52]. These tRFs mostly involved in protein phosphorylation, transcription, cell migration, the pathway involved in cancer, and the MAPK/Wnt signaling pathways. Among them, tRF-03357 was upregulated in HGSOC serum samples and SK-OV-3 cells. Furthermore, tRF-03357 promoted the proliferation, migration, and invasion of SK-OV-3 cells by modulating the transcription factor HMBOX1. In addition to tRF-03357, one tRF derived from tRNA-Glu was identified in ovarian cancer. tRNA-Glu-CTC (tRF5-Glu) was confirmed to bind directly to a site in the 3′UTR of the Breast Cancer Anti-Estrogen Resistance 3 (BCAR3) mRNA and hence reduce its expression, ultimately inhibiting the proliferation of ovarian cancer cells [53].

After transfection with a tRF-Leu-CAG inhibitor, the proliferation capacity of lung cancer cells decreased, the number of cells in the G0/G1 phase increased, and the expression of auroral kinase A (AURKA) protein increased significantly [45]. AURKA is a highly conserved serine kinase/threonine kinase that participates in the control of the cell cycle and is related to the regulation of some cell division-related biological processes [54]. Previous studies have shown that miRNA-137 and miRNA-32 can affect the progression of NSCLC by acting on AURKA [54, 55].

CU1276 is a representative tRF-3 that is overexpressed in mature B cells but repressed in lymphoma cell lines [40]. Stable expression of CU1276 in Burkitt lymphoma cells can inhibit cell proliferation, and when exogenous RPA1 is coexpressed, the proliferation of lymphoma cells can be restored. Similarly, the strong expression of CU1276 leads lymphoma cells to become more sensitive to drug-induced DNA damage. Restoration of RPA1 levels impeded this sensitization. These results suggested that CU176 inhibits lymphoma cell proliferation and is involved in DNA damage in an RPA1-dependent manner [40, 56].

Moreover, there is evidence suggesting that tRFs and tiRNAs may regulate gene expression by binding to RBPs. tRFs from tRNA-Asp, tRNA-Glu, tRNA-Tyr, and tRNA-Gly can suppress cell growth under serum-starvation conditions by binding to YBX1, an RBP with multiple biological functions, and repressing its combination with some endogenous oncogene transcripts in breast cancer [57].

### Cell apoptosis

The resistance to apoptosis, or programmed cell death, is another important trait that allows cancer cells to expand their population and escape drug-induced extinction. Since tsRNAs are derived from tRNA, it can be inferred that tsRNAs may share some functions with tRNAs. Previous evidence suggested that tRNAs inhibit apoptosis by binding to cytochrome C (Cyt c) [58]. Saikia et al. found that Cyt c could directly bind to tiRNAs to form a ribonucleoprotein complex, block the oligomerization of Apaf-1 to reduce the formation or activity of apoptotic bodies, and finally, inhibit apoptosis [59]. In contrast, 5′-tiRNA-Glu can induce endonuclease tRNase ZL to cleave target mRNAs in vivo and in vitro to regulate apoptosis [60]. Protein phosphatase 1F (PPM1F) mRNA is one of the target mRNAs of tRNaseZL, and its overexpression in HeLa cells can induce apoptosis [60]. In comparison, Mo et al. found that the survival rate and colony formation of breast cancer cells transfected with 5′-tiRNA-Val were significantly lower than those of negative controls. Subsequent experiments confirmed that 5′-tiRNA-Val targets to the human Frizzled homologous gene 3 (FZD3) and attenuates cell proliferation, invasion, and metastasis by inhibiting the Wnt/β-catenin signal pathway [47].

tsRNA-Leu-CAG 3′ could bind to at least two ribosomal protein mRNAs RPS28 and RPS15, and then promote their translation. Kim et al. found that tsRNA-Leu-CAG 3′ inhibition suppressed RPS28 translation which hindered the processing of 18S, and 40S was reduced [61]. Finally, the apoptosis of hepatoma cells was accelerated after tsRNA-Leu-CAG 3′ inhibition.

**Table 2:** Applications of tsRNAs in cancer diseases.

	tsRNA	Cancer type	Characteristics	Reference
Single tsRNAs as biomarkers or therapeutic targets	tRNA-ValTAC-3	Liver cancer	All upregulating	[76]
	tRNA-Gly-TCC-5			
	tRNA-Val-AAC-5			
	tRNA-Glu-CTC-5			
	tRF-Leu-CAG	NSCLC	Upregulating	[45]
	tRF-30-JZOYJE22RR33	Breast cancer	Upregulating	[77]
	tRF-27-ZDXPHO53KSN			
	tDR-0009 tDR-7336	TNBC	Upregulating	[95]
	tRF-32-XSXMSL73VL4YK tRF-32-Q99P9P9NH57SJ tRF-17-79MP9PP	Breast cancer	Upregulating	[96]
			Downregulating	
			Downregulating	
	miR-21-5p miR-23a-3p tRF-Lys	Breast cancer	All upregulating	[97]
	tRF-Glu-CTC-003 tRF-Gly-CCC-007 tRF-Gly-CCC-008 tRF-Leu-CAA-003 tRF-Ser-TGA-001 tRF-Ser-TGA-002	Breast cancer	All downregulating	[98]
	ts-34 ts-49	Breast cancer	Low level of ts-34 or a high level of ts-49 showed improved survival	[99]
	miR-122-5p miR-142-3p 5′tRNA4-Val-AAC	Clear cell renal cell carcinoma	Upregulating	[100]
			Upregulating	
			Downregulating	
Multiple tsRNA expression signatures as risk factors	tRNALysCTT tRNAPheGAA	Prostate cancer	Upregulating	[101]
			Downregulating	

### Cell migration and tumor metastasis

Metastasis is the main characteristic of malignant tumors, and it is a critical hallmark of tumor progression and poor prognosis. Uveal melanoma (UVM) is an intraocular malignant tumor with a metastatic rate of over 50%. After an analysis of 80 UVM samples, the expression of tRFs showed significant differences in the metastatic samples [62]. Samples with metastasis had a higher proportion of 18 nt tRFs and a lower proportion of 20 nt tRFs. In addition, M3 patients showed a higher expression of 18 nt tRFs [62]. A previous study found that by binding to the primer binding site of long terminal repeat-retrotransposons, 18-nt-long tRFs hinder reverse transcription and retrotransposon mobility, while 22-nt-long tRFs prevent transposon expression [63]. These results suggested that tRFs involved in tumor metastasis and tRFs of different lengths may have distinct functions in this malignant process.

One ncRNA called tRF/miR-1280 that is decreased in colorectal cancer tissues derived from both tRNA-Leu and pre-miRNA was identified as a regulator of colorectal cancer [64]. Huang et al. colleagues revealed that the overexpression of tRF/miR-1280 significantly inhibited the migration and mobility of colorectal cancer cells by preventing premetastatic niche (PMN) formation. It is widely believed that the PMN initiates angiogenesis and remodeling of the stroma and extracellular matrix [65]. In addition, Huang’s results showed that tRF/miR-1280 overexpression antagonized PMN formation by reducing the expression of CD31, MMP-2, and MMP-9. The endothelial–mesenchymal transition (EMT) is a malignant process and plays a critical role in the carcinogenesis and metastasis of cancers [66]. Recently, it was reported that tRF-20-M0NK5Y93 inhibited the EMT of colorectal cancer cells by targeting Claudin-1, thereby regulating the migration and invasion of colorectal cancer cells [67]. In another study, tRF-20-MEJB5Y13 was identified to be elevated in colorectal cancer under hypoxic conditions. This Dicer1-induced overexpression was demonstrated to be responsible for hypoxia-induced colorectal cancer cell invasion and migration [68].

Meanwhile, the metastasis regulatory functions of tsRNAs are also found in other malignancies. tRF-3017A was found to silence the tumor suppressor NELL2 through an AGO-dependent mechanism, thereby promoting the invasion and migration of gastric cancer cells [69]. tRF-17 was found to act as an invasion inhibitor by regulating the THBS1/TGF-β1/smad3 axis in breast cancer cells [70].

### Tumor microenvironment

A growing body of evidence has demonstrated that tumor cells can produce extracellular vesicles such as microvesicles (EVs) and exosomes to communicate with other stromal cells by the transportation of ncRNAs and short-chain polypeptides [71]. This biological process can reshape the tumor microenvironment and facilitate tumor growth [72]. Recent studies have shown that EVs derived from cancer cells also include tRFs. In 2013, researchers reported the existence of tRNAs in human plasma exosomes for the first time [73]. A few years later, tRFs were found in EVs from breast cancer cells, glioma stem cells, and liver cancer cells [74–76]. These results suggested that tRFs may have critical roles in tumor microenvironment communication. However, no significant biological function of these exosome-derived tRFs has been identified thus far. Whether these tRFs in EVs can regulate angiogenesis, fibroblasts, or the immune environment of tumor cells remains to be determined.

### Drug resistance

Drug resistance capacity is another important characteristic of cancer cells. There is emerging evidence implying that tRFs may be novel tumor drug resistance regulatory factors. Sun et al. demonstrated that tRF-30-JZOYJE22RR33 and tRF-27-ZDXPHO53KSN were correlated with trastuzumab resistance in breast cancer [77]. Another bioinformatic analysis showed that tDR-0009 (tDR-7336) might be involved in the chemoresistance and doxorubicin resistance of triple-negative breast cancer (TNBC) via regulation of STAT3 phosphorylation and the IL-6 response [78]. Moreover, tRF-315 derived from tRNA-Lys was demonstrated to be overexpressed in prostate cancer cells and relieved the cisplatin-induced apoptosis and mitochondrial dysfunction of these cancer cells by targeting the cell cycle-related gene GADD45A [79]. Therefore, research on the drug resistance functions of tRFs in cancer cells is in its early stages, and there will be more studies that reveal the mechanisms of tRF regulation of cancer drug resistance in the future.

### Cancer stem cells

In the last decade, cancer stem cells (CSCs) have attracted increasing attention and become a new emerging hallmark of cancers. CSCs are a group of highly tumorigenic cancer cells that participate in many malignant biological functions such as EMT, tumor proliferation, and especially drug resistance and tumor recurrence [80]. In recent years, tRNA fragments have been proven to regulate protein translation and cell differentiation in somatic stem cells [32, 81]. Moreover, tRNA fragments were found to be critical for CSC functions in recent studies. tRF/miR-1280 was proven to reduce cell proliferation and colony formation and suppress CSC phenotypes via Notch signaling in colorectal cancer [64]. Wei et al. identified GluCTC and GlyCCC tRNA fragments that were enriched in microvesicles and exosomes from human glioma stem cells [75]. However, the molecular mechanisms of these two small RNAs in CSC functional regulation have barely been discussed.

### Cancer metabolism

Energy metabolism in cancer cells is another noticeable hallmark of cancer disease. Recent studies reported that cancer cells can reprogram their glucose metabolism by suppressing the tricarboxylic acid cycle and enhancing glycolysis in the presence of oxygen, so-called “aerobic glycolysis” [82]. For the past few years, evidence has implied that tRNA fragments may have regulatory roles in the metabolic alterations of cancer cells. Researchers analyzed isoforms of miRNAs and tRNA fragments in TNBC and found that most dysregulated sncRNA-related mRNAs were enriched in oxidative phosphorylation and ribosome biogenesis [83]. One year later, another study published in the same journal showed that abnormally expressed tRF-related mRNAs were also correlated with glycolysis and ATP synthesis in almost all solid tumor types [84]. Moreover, other studies have also suggested the role of tRFs in protein biosynthesis in normal or tumor cells [32, 85].

In summary, although there is some evidence demonstrating the vital role of tRFs in metabolic alterations in cancer cells, deeper mechanistic insight into tRNA fragment regulation in this cancer hallmark are is needed.

## Applications of tsRNAs in cancer diseases

### Single tsRNAs as biomarkers or therapeutic targets of tumors

Early diagnosis of cancer is the key to obtaining improved treatment reactivity and prognosis. A growing body of evidence has demonstrated that miRNAs, cyclic RNAs, and long noncoding RNAs in exosomes have shown great potential as biomarkers for the diagnosis or prognosis of malignant diseases [86]. Recent studies have identified tsRNAs as a new kind of tumor biomarker, other ncRNAs and circulating tumor DNAs (ctDNAs) (Table 2). The content of serum tsRNAs in liver cancer patients was significantly higher than that in healthy donors. Among the tsRNAs, tRNA-ValTAC-3, tRNA-Gly-TCC-5, tRNA-Val-AAC-5, and tRNA-Glu-CTC-5 were the most significantly elevated [76]. Similarly, tRF-Leu-CAG was proven to be a potential marker for NSCLC, because it is significantly upregulated in NSCLC tissues, cell lines, and serum [45]. After screening differentially expressed tRNAs between lung cancer tissues and paracarcinoma tissues, Kuang et al. demonstrated that tRNA-Asn-ATT, tRNA-Ile-AAT, tRNA-Leu-TAA, mt-tRNA-Trp-TCA, mt-tRNA-Leu-TAA, tRNA-Pro-AGG, tRNA-Lys-CTT-1, and tRNA-Leu-AAG were associated with the clinicopathological characteristics of lung adenocarcinoma. Among them, tRNA-Lys-CTT-1, mt-tRNA-Ser-GCT, and tRNA-Tyr-ATA were associated with cancer-specific survival [87]. These specifically expressed tRFs can be used to construct prognostic models for lung cancer patients. Sun et al. found that tRF-30-JZOYJE22RR33 and tRF-27-ZDXPHO53KSN were highly expressed in trastuzumab-resistant breast cancer cells compared to trastuzumab-sensitive breast cancer cells [77]. Furthermore, the high levels of these two tRFs were related to shorter progression-free survival (PFS) of HER-2 positive breast cancer patients. These results indicated that tRF-30-JZOYJE22RR33 and tRF-27-ZDXPHO53KSN can be biomarkers and novel therapeutic targets for HER-2 positive breast cancer patients with trastuzumab resistance. Recently, researchers also found tRFs that act as biomarkers for the diagnosis and prognostic prediction in gastric cancer and myeloma [88–91].

### tsRNA expression signatures as risk factors for cancer patients

With the development of bioinformatic technologies, researchers can combine the expression levels of multiple genes to construct a new model that can predict the prognosis of cancer patients. According to different gene set selections, this study strategy has been applied to many malignancies such as glioma, gastric cancer, and lung cancer [92–94]. With the development of tsRNA-related research, expression information for multiple tsRNAs can also be organized as a gene group to construct models. To date, most studies have focused on the discovery of abnormal expression of multiple tsRNAs in breast cancer. tDR-0009 and tDR-7336 were significantly upregulated in TNBC cells. Another study revealed that tRF-32-XSXMSL73VL4YK was significantly upregulated, and tRF-32-Q99P9P9NH57SJ and tRF-17-79MP9PP were significantly downregulated in breast cancer tissues [95, 96]. Kio and his colleagues combined the miRNA and tsRNA pools and found that the expression of miR-21-5p, miR-23a-3p, and tRF-Lys, was significantly higher in breast cancer samples and these results could be validated in an independent cohort by small RNA sequencing [97]. Circulating tsRNAs have also received attention. Six tsRNAs (tRF-Glu-CTC-003, tRF-Gly-CCC-007, tRF-Gly-CCC-008, tRF-Leu-CAA-003, tRF-Ser-TGA-001, and tRF-Ser-TGA-002) were found significantly downregulated in plasma samples of patients with early breast cancer compared with normal controls [98]. Another study showed that specific tRF (ts-34 and ts-49) signatures were associated with a single immune-related pathway (T cell activation) in BC cells [99]. Therefore, it can be imagined that a specific tRF signature will soon be constructed to predict the prognosis of breast cancer patients.

In addition to breast cancer, there are also studies indicating multiple tsRNA signatures in other malignancies such as papillary thyroid cancer, pancreatic cancer, and colon cancer. Nientiedt and his colleagues also pooled miRNAs and tsRNAs together as sncRNAs and discovered that miR-122-5p, miR-142-3p, and 5′tRNA4-Val-AAC were abnormally expressed in clear cell renal cell carcinoma tissues, but not in the serum from patients [100]. To date, only one multiple tRF signature has been identified in prostate cancer. The authors showed that the ratio of tsRNAs derived from tRNALysCTT and tRNAPheGAA directly predicted PFS and could be a candidate prognostic marker for prostate cancer patients [101]. In the future, more high-quality studies will construct more tRF signatures to predict other kinds of cancers.

## Conclusion and perspectives

tsRNAs are a novel group of sncRNAs and have critical roles in the development of most malignancies. They have been proven to regulate proliferation, apoptosis, migration, CSC phenotypes, and other vital cancer hallmarks. Meanwhile, emerging evidence implies that tsRNAs may participate in the remodeling of the tumor microenvironment and control the metabolism and drug resistance capacity of cancer cells. Moreover, the application of tsRNAs as cancer biomarker candidates and therapeutic targets is starting to receive more attention. Therefore, tsRNA-related biological regulation in cancer disease will undoubtedly become a popular research topic in the future. However, there are also challenges to performing in-depth research on this regulatory network.

First, the database of tsRNAs should be perfected. Although some online tsRNA databases can provide the sequence and expression level of tsRNAs in tumors, such as OncotRF, tRFexplorer, and tRFdb, the nomenclature of tsRNAs has not been standardized [6, 26, 102]. Therefore, the search for tsRNAs in the literature database is difficult and inefficient. Moreover, complementary pairing and target prediction information should be added to the database because it is important for research on miRNA-like mechanisms. Meanwhile, the molecular mechanisms of tsRNA regulation remain to be explored. tsRNAs can function as ncRNAs like miRNAs, but they exhibit much more complicated regulatory mechanisms. tsRNAs regulatory patterns remain in case report-like summaries, especially RBP-related functions. In the future, the general pattern of tsRNA regulation should be summarized.

Given the difficulties mentioned above, most of the current tsRNA-related studies are superficial in terms of describing the detailed mechanisms involved in cancer hallmark regulation. Therefore, with novel understanding of tsRNA regulatory mechanisms, more in-depth research on cancer hallmark regulation by tsRNAs can provide an innovative view for the comprehension of cancer cell biology.

## Supplementary information

author contribution form

author contribution form

## Data Availability

All data included in this study are available upon request by contact with the corresponding author.
