# Supplementary figures and images for: tRNA-derived small RNAs: novel regulators of cancer hallmarks and targets of clinical application
Source: Cell Death Discov. 2021 Sep 18;7:249. doi: 10.1038/s41420-021-00647-1 (PMC8449783; doi:10.1038/s41420-021-00647-1)

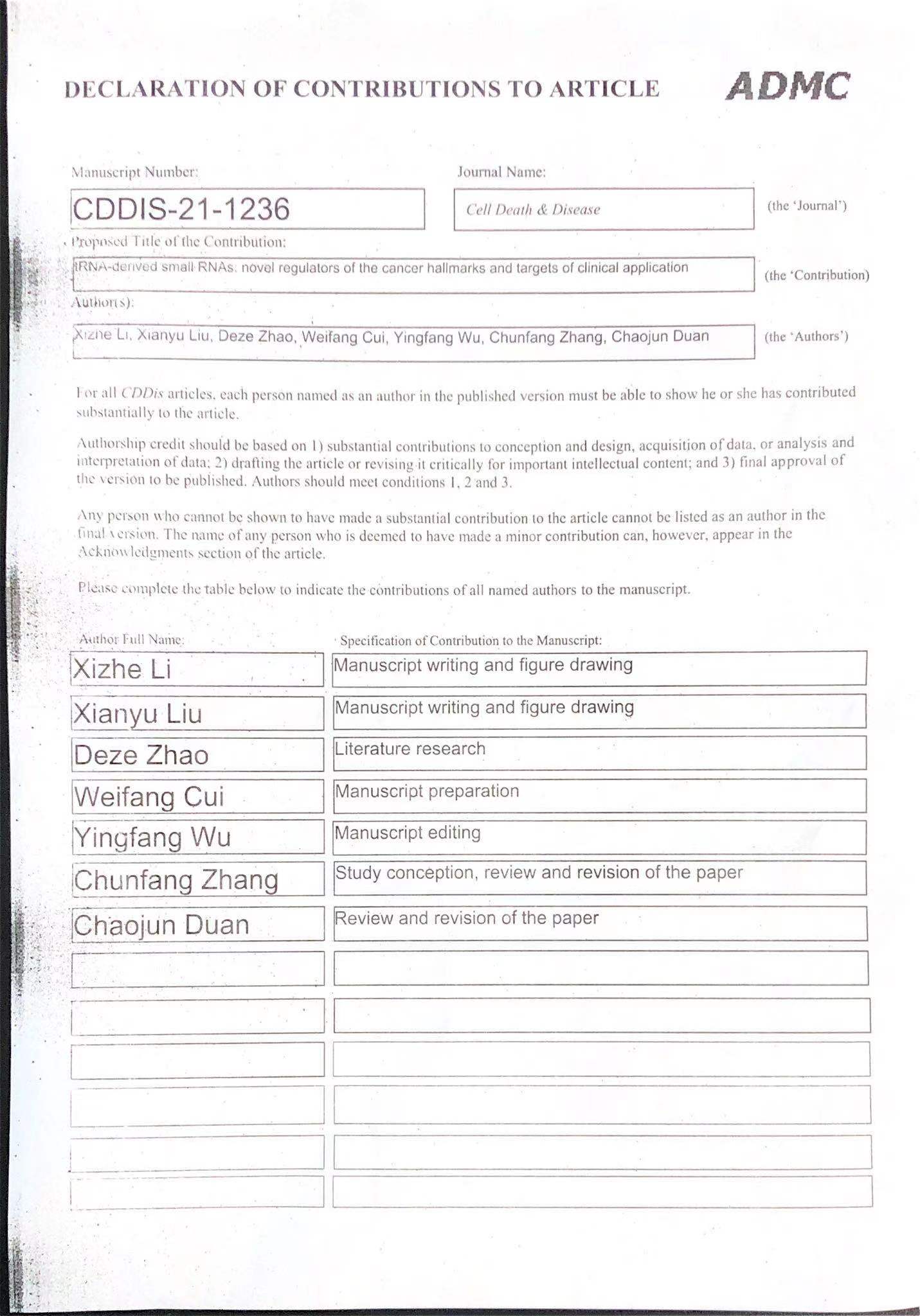

Supplement: Supplementary file 1 — author contribution form [file 41420_2021_647_MOESM1_ESM.jpg]

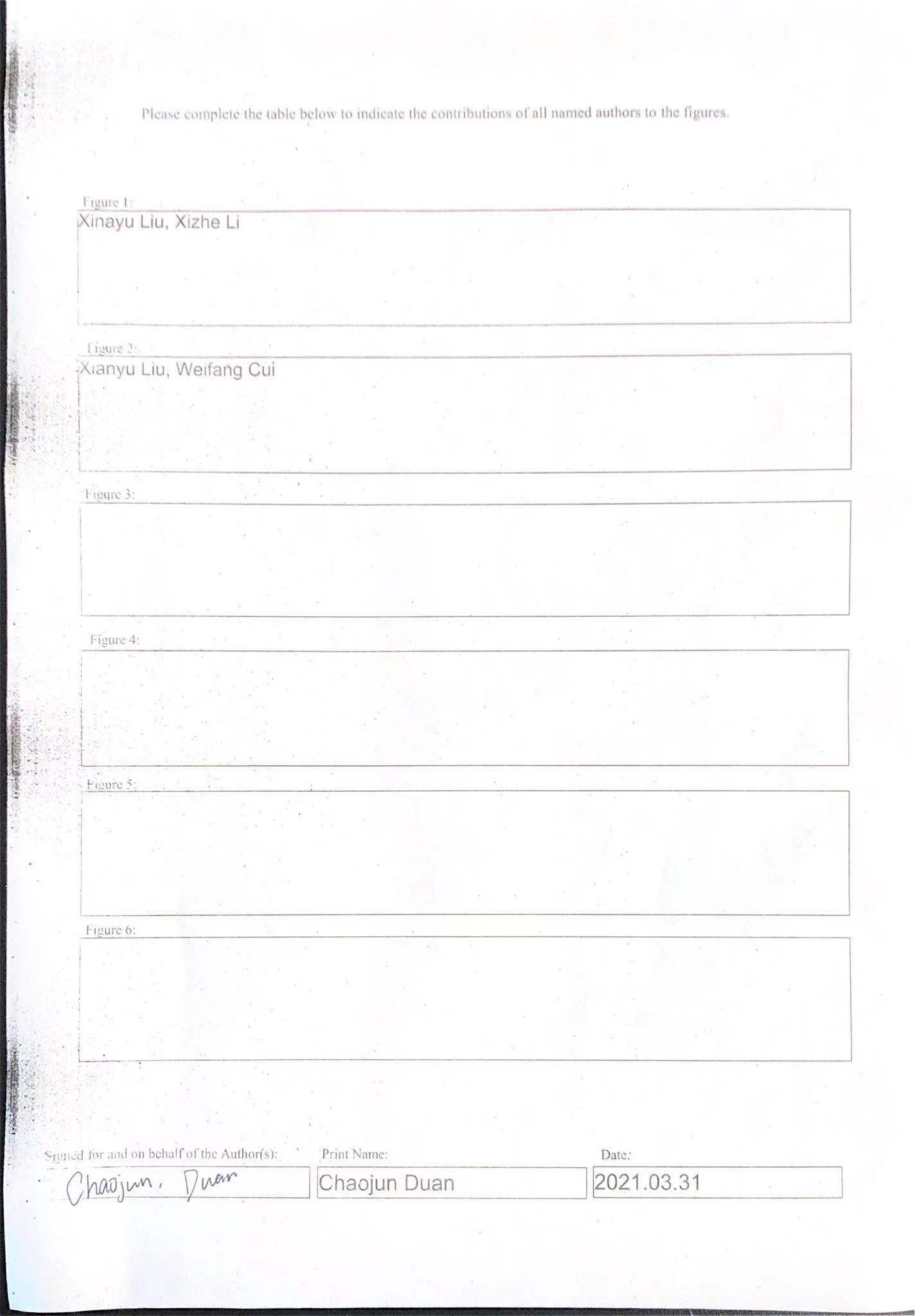

Supplement: Supplementary file 2 — author contribution form [file 41420_2021_647_MOESM2_ESM.jpg]
